# Wound infection in colorectal cancer resections through a laparoscopic approach: a single-center prospective observational study of over 3000 cases

**DOI:** 10.1007/s12672-021-00396-8

**Published:** 2021-02-11

**Authors:** Atsushi Ikeda, Yosuke Fukunaga, Takashi Akiyoshi, Satoshi Nagayama, Toshiya Nagasaki, Tomohiro Yamaguchi, Toshiki Mukai, Yukiharu Hiyoshi, Tsuyoshi Konishi

**Affiliations:** 1 Department of Gastroenterological Surgery, Cancer Institute Hospital of Japanese Foundation for Cancer Research, Tokyo, Japan; 2 Department of Surgery, Graduate School of Medicine, Kyoto University, Kyoto, Japan; 3 Department of Surgical Oncology, The University of Texas, M.D. Anderson Cancer Center, 1400 Pressler Street Unit 1484, Houston, TX 77030 USA

## Abstract

**Objectives:**

This prospective observational study aimed to clarify the incidence and independent risk factors of wound infection after laparoscopic surgery for primary colonic and rectal cancer.

**Methods:**

A prospective surveillance of surgical site infection (SSI) was conducted in consecutive patients with primary colorectal cancer, who underwent elective laparoscopic surgery in a single comprehensive cancer center between 2005 and 2014. The outcomes of interest were the incidence and risk factors of wound infection.

**Results:**

In total, 3170 patients were enrolled in the study. The overall incidence of wound infection was 3.0%. The incidence of wound infection was significantly higher in rectal surgery than in colonic surgery (4.7 vs. 2.1%, *p* < 0.001). In rectal surgery, independent risk factors for developing wound infection included abdominoperineal resection (*p* < 0.001, odds ratio [OR] = 11.4, 95% confidence interval [CI]: 5.04–24.8), body mass index (BMI) ≥ 25 kg/m^2^ (*p* = 0.041, OR = 1.97, 95% CI, 1.03–3.76), and chemoradiotherapy (*p* = 0.032, OR = 2.18, 95% CI, 1.07–4.45). In laparoscopic colonic surgery, no significant risk factors were identified.

**Conclusions:**

Laparoscopic rectal surgery has a higher risk of wound infection than colonic surgery. Laparoscopic rectal surgery involving abdominoperineal resection, patients with higher BMI, and chemoradiotherapy requires careful observation in wound care and countermeasures against wound infection.

## Introduction

Wound infection is one of the most frequent types of nosocomial infections, and it is a major cause of postoperative complications. Particularly in colorectal surgery, wound infection develops at a higher rate than in other types of operations [1], due to the high bacterial load present within the colorectal lumen. Although wound infection is rarely life-threatening, it occasionally leads to mortality. In addition, medical cost increases, hospital stay is prolonged, and the patient’s quality of life decreases [2, 3]. Therefore, prevention against wound infection has been regarded as one of the most important issues for colorectal surgeons [4, 5]. In open procedures, rectal surgery has different incidence rates and risk factors of wound infection, compared with colon surgery [6, 7].

Since its introduction in 1991 [8], laparoscopic colorectal surgery has been performed at an increasing number of institutes, and many studies have demonstrated its advantage with regard to wound infection [9–12]. Such studies have also evaluated the risk factors for wound infection after laparoscopic colorectal surgery [10, 13–16]. However, few studies with large sample sizes have focused on the differences in wound infection between laparoscopic colon and rectal cancer resections. Considering the higher technical demand in laparoscopic rectal cancer surgery compared with colonic, these two laparoscopic procedures might have different profiles that require different wound management approaches.

This study aimed to evaluate the differences in incidence rates and risk factors of wound infection between laparoscopic colon and rectal surgeries for primary colorectal cancer, using data from a prospective surveillance program of surgical site infection (SSI) at a single comprehensive cancer center.

## Methods

### Surveillance methods and patient population

From July 2005 to February 2014, all patients undergoing elective laparoscopic colorectal surgery for primary colorectal cancer were enrolled into a prospective SSI surveillance program at a single comprehensive cancer center in Japan. We prospectively collected the surveillance data, including the patients’ demographics, surgical characteristics, and surgical outcomes with postoperative complications [14–21]. The primary outcome of interest was the incidence of wound infection, and the secondary outcome of interest was the independent risk factors of wound infection. Our SSI surveillance system was based on the U.S National Nosocomial Infections Surveillance (NNIS) system [22], and all the data were prospectively collected for at least 30 days after surgery.

SSI, including wound infection, was defined according to the guidelines issued by the Center for Disease Control and Prevention [23]. Briefly, the criteria for incisional SSI were as follows: an infection occurring at the incision site within 30 days after the surgery, involving the skin, subcutaneous tissue, muscle, and fascia but not organ space, and at least one of the following: purulent drainage from the incision; an organism isolated from a culture of fluid from the incision; incisional pain, tenderness, localized swelling, redness, or heat; an incision that spontaneously dehisced or was deliberately opened by a surgeon considering the signs and symptoms of infection described previously. Superficial and deep incisional SSIs were evaluated together under the umbrella term “wound infection.” Patients were assessed daily for SSI until discharge by attending physicians and nurses, with a follow-up of at least 30 days postoperatively in the outpatient clinic.

Colonic surgery was defined as procedures with anastomosis carried out above the peritoneal reflection, while rectal surgery was defined as those carried out below. Operative procedures were categorized as right colonic surgery, left colonic surgery, low anterior resection, intersphincteric resection, abdominoperineal resection (APR), and Hartmann's procedure. All procedures were performed or supervised by seven expert colorectal surgeons, each of whom had performed over 500 laparoscopic colorectal procedures and had a board-certification for endoscopic surgery from the Japan Society for Endoscopic Surgery.

### Preoperative protocols and surgical procedures

All patients preoperatively underwent mechanical bowel preparation with oral laxative and magnesium citrate on the day before surgery, except in the cases of bowel obstruction by the tumor [24]. All patients also underwent chemical bowel preparation with 1 g of metronidazole and 750 mg of kanamycin. For systemic antimicrobial prophylaxis, 1 g of cefmetazole was administered 30 min before the skin incision, repeated every 3 h during the operation, and stopped within 24 h after the surgery. Preoperatively, the patient’s hair on the surgical field was shaved using electrical clippers on the day before surgery. After the induction of general anesthesia, the surgical site was wiped with povidone iodine solution and then covered with disposable drapes. Abdominal incisions were closed primarily with polydioxanone (PDS) monofilament absorbable sutures for both the fascia and skin. Subcutaneous drains were not used.

### Dependent and independent variables

The outcome of interest was the incidence of wound infection. All of the independent variables were evaluated as categorical variables, including patient age in years (≤ 60, 61–70, ≥ 71), sex, body mass index (BMI) in kg/m^2^ (< 25, ≥ 25), American Society of Anesthesiologists (ASA) score (1, 2, ≥ 3), presence of diabetes mellitus, tumor, node, metastasis (TNM) stage (I, II, III, IV, carcinoma in situ or adenoma, other types of tumor), operative procedure (right colonic surgery, left colonic surgery, low anterior resection, intersphincteric resection, APR, Hartmann's procedure), ostomy creation, duration of operation in hours (< 3, 3–4, ≥ 4), blood loss in mL (< 100, ≥ 100), and preoperative chemoradiotherapy. Other variables were categorical and are presented in the results.

### Statistical analysis

Statistical analysis was performed using JMP software (version 14.0, SAS Institute Inc., Cary, NC, USA). The univariate relationships between each independent variable and wound infection were evaluated using Pearson’s χ^2^ test or Fisher’s exact test, as appropriate. Only variables with a *p* value of less than 0.2 in the univariate analysis were included in a multivariate model of logistic regression, and the odds ratio, with 95% confidence intervals for each variable, were determined. *P* values less than 0.05 were considered statistically significant.

## Results

In total, 3170 patients underwent elective laparoscopic colorectal cancer surgery during the 106-month period, and all patients were admitted to our surveillance program, with all cases being eligible for the study. Of the total number of patients, 95 (3.0%) were diagnosed with wound infection. Of all 3170 cases, 2037 (64%) underwent colonic surgery and 1133 (36%) underwent rectal surgery.

Table 1 shows the association between patient characteristics and wound infection after colonic and rectal surgeries. In univariate analysis, BMI and ASA score were statistically associated with the development of wound infection in rectal surgery (*p* = 0.001 and 0.016, respectively). However, none of the variables were statistically associated with wound infection in colonic surgery.

**Table 1:** Patient characteristics and wound infection

Variables	Colon (n = 2037)			Rectum (n = 1133)		
	n	Wound infection (%)	*p*	n	Wound infection (%)	*p*
Age (year)			0.471			0.547
≤ 60	671	1.9		551	4.4	
61–70	700	2.6		350	4.3	
≥ 71	666	1.7		232	6.0	
Gender			0.589			0.295
Female	983	2.2		463	3.9	
Male	1054	1.9		670	5.2	
BMI (kg/m^2^)			0.939			*0.001*
< 25	1562	2.1		837	3.5	
≥ 25	475	2.1		296	8.1	
ASA score			0.596			*0.016*
1	634	2.2		446	2.9	
2	1357	2.1		664	5.6	
≥ 3	46	0		22	13.6	
Diabetes mellitus			0.729			0.205
+	115	0.9		61	8.2	
−	1922	2.1		1072	4.5	
TNM stage			0.752			0.901
I	626	1.6		416	4.1	
II	513	2.3		221	5.9	
III	560	2.3		338	5.0	
IV	199	1.5		60	3.3	
Carcinoma in situ or adenoma	121	3.3		42	4.8	
Other types of cancer	18	0		56	3.6	

Italic signifies *p* < 0.05

Table 2 shows the association between surgical characteristics and wound infection. In univariate analysis, surgical procedures, ostomy creation, duration of operation, blood loss, and chemoradiotherapy had significant correlations with the development of wound infection in rectal surgery (*p* < 0.001, *p* < 0.001, *p* = 0.004, *p* = 0.002, *p* < 0.001, respectively). However, in colonic surgery, none of the variables associated statistically with the development of wound infection.

**Table 2:** Surgical characteristics and wound infection

Variable	Colon (n = 2037)			Rectum (n = 1133)		
	No	Wound infection (%)	*p*	No	Wound infection (%)	*p*
Procedures						
Colon surgery			0.566			
Right colectomy	830	1.7				
Left colectomy	1201	2.3				
Hartmann’s procedure	6	0				
Rectal surgery						<* 0.001*
Low anterior resection				825	2.1	
Intersphicteric resection				165	0.6	
Abdominoperineal resection				133	25.6	
Hartmann’s procedure				10	10.0	
Ostomy creation			1.000			< *0.001*
+	8	0		598	7.2	
−	2029	2.1		535	1.9	
Duration of operation (hours)			0.579			*0.004*
< 3	733	1.6		107	1.9	
3–4	797	2.4		330	2.1	
≥ 4	507	2.2		696	6.3	
Blood loss			0.512			*0.002*
< 100 ml	1917	2.1		944	3.7	
≥ 100 ml	116	0.9		189	9.5	
Chemoradiotherapy						< *0.001*
+	0	0		269	10.8	
−	2030	2.1		864	2.8	

Italic signifies *p* < 0.05

Table 3 summarizes the results of the multivariate logistic regression analysis. BMI, operative procedure (APR), and chemoradiotherapy were the independent risk factors for the development of wound infection after rectal surgery (BMI: OR = 1.97, 95% CI = 1.03–3.76, *p* = 0.041, operative procedure [APR]: OR = 11.2, 95% CI = 5.04–24.8, *p* < 0.001, and chemoradiotherapy: OR = 2.18, 95% CI = 1.07–4.45, *p* = 0.032).

**Table 3:** Multivariate logistic regression analysis for risk factors of wound infection in rectal surgery

Variable	Odds ratio	95% CI	*p*
BMI ≥ 25	1.97	1.03–3.76	*0.041*
ASA			
1		Reference value	
2	1.85	0.90–3.80	0.096
≥ 3	4.90	0.92–26.2	0.063
Operation			
Low anterior resection		Reference value	
Intersphicteric resection	0.21	0.03–1.71	0.146
Abdominoperineal resection	11.2	5.04–24.8	< *0.001*
Hartmann’s procedure	3.57	0.37–34.1	0.268
Ostomy creation	1.12	0.43–2.90	0.146
Duration of operation			
< 3		Reference value	
3–4	0.79	0.15–4.10	0.780
≥ 4	1.44	0.31–6.60	0.642
Blood loss			
≥ 100 ml	0.78	0.38–1.61	0.507
Chemoradiaotherapy	2.18	1.07–4.45	*0.032*

Italic signifies *p* < 0.05

*ASA* American Society of Anesthesiologists, *BMI* body mass index, Other types of tumors include carcinoids and gastrointestinal stromal tumors

## Discussion

This study found a higher incidence of wound infection in laparoscopic rectal resection than in colon resection and identified the risk factors for developing wound infection in a prospective dataset that included more than 3000 patients with colorectal cancer in a single cancer center. This is one of the largest series of SSI surveillance ever reported in the field of laparoscopic surgery for colorectal cancer [25, 26]. In this prospective single-center study, the surgical procedures and perioperative managements were conducted under the unified protocol without variance among the providers, which possibly minimized the biases related to the different perioperative care among the providers, in contrast to the previous studies [25, 26].

In the present study, the incidence rates of wound infection were 2.1% (42/2037) in laparoscopic colonic surgery and 4.7% (53/1133) in laparoscopic rectal surgery, which is acceptable in comparison with the results of previous studies reported so far [11, 27, 28]. During the study period, the incidence of wound infection in elective open resections for primary colonic and rectal cancers were 4.3% (17/392) and 17.5% (28/160), respectively. Compared to these data, the present study clearly demonstrated that a laparoscopic approach was associated with lower rates of wound infection in colon and rectal procedures (*p* = 0.012 and < 0.001, respectively). Consistent with the results of previous studies [27], our findings confirmed the advantage of laparoscopic surgery in reducing wound infection in both colonic and rectal cancer surgeries. In laparoscopic colonic surgery, the outcome events are so rare that no risk factor can be found in a study even with such a large number of cases. In other words, with sophisticated surgical techniques and well-trained perioperative management, wound infection after laparoscopic colonic surgery can be reduced to minimal incidence. In contrast, after laparoscopic rectal surgery, BMI > 25 kg/m^2^, operative procedure (APR), and chemoradiotherapy were found to be independent risk factors for the development of wound infection, which suggests the need for careful observation in wound care and countermeasures against wound infection in these patients.

Many studies have investigated the relationship between obesity and wound infection in laparoscopic colorectal surgery [29–31]. Some studies have demonstrated a significant association between obesity and wound infection, which might be explained by increased fractions of visceral fat, higher incidence of metabolic syndrome, and a narrow pelvis [30]. In the present study, the incidence of wound infection after laparoscopic colonic surgery was similar between patients with higher and lower BMI. In rectal surgery, however, wound infection developed with a significantly higher rate in patients with higher BMI. Laparoscopic rectal surgery in obese patients is particularly challenging because of the difficulty in accessing the pelvic operative field, and high BMI is reportedly associated with a higher incidence of postoperative complications [32–34]. Attention is required in perioperative wound care after laparoscopic rectal surgery in patients with higher BMI.

APR is reportedly associated with a high incidence of perineal wound complications, up to 58% [35]. After the rectum is excised, the sacral cavity forms a large wound area, which leads to fluid collection and results in pelvic abscess. Similarly, in the present study, laparoscopic APR was associated with a higher incidence of wound infection, compared with other procedures. Interestingly, patients who underwent open APR during the same period in our institute developed wound infection with a rate of 31.5% (17/54), which is similar to the rate in laparoscopic APR (25.6%) in this study. The perineal procedure in APR is the same, regardless of open or laparoscopic approaches, which likely eliminates the benefit of the laparoscopic approach in terms of reducing wound infection. APR often requires longer operative time than other colorectal resections due to the time-consuming perineal procedure [36], which further increases the risk of wound infection. The incidence of wound infection in the perineal wound was further elevated by chemoradiotherapy [37], and 33.3% (25/75) of the patients who underwent laparoscopic APR after chemoradiotherapy developed wound infection in this study. In light of these findings, management of the perineal wound in APR is a matter of concern in laparoscopic surgery and requires further improvement.

The strengths of this study include prospective surveillance data with a large sample size, unified perioperative management under a predefined protocol, and a study cohort that focused on laparoscopic surgery for colorectal cancer at a comprehensive cancer center. However, the study has limitations inherent to a single center study. Although we identified higher BMI as a risk factor for wound infection in rectal cancer, data from a specialty institution in Japan, where patients are generally fit and non-obese, have the potential for limited applicability. The generalizability of the present findings should be validated in different cohorts with higher BMI values. Despite these limitations, our findings clearly identified different profiles of wound infection between laparoscopic colon and rectal cancer resections. Further studies are required to reveal the need for different wound management approaches in these procedures.

In conclusion, laparoscopic rectal surgery has a higher risk of wound infection, compared with laparoscopic colonic surgery, with distinct profiles of the risk factors. Laparoscopic rectal surgery involving abdominoperineal resection, patients with higher BMI, and chemoradiotherapy requires careful observation in wound care and countermeasures against wound infection.

## Data Availability

The datasets generated and analyzed in this study are available from the corresponding author upon reasonable request.
